# A comparative analysis of rumen pH, milk production characteristics, and blood metabolites of Holstein cattle fed different forage levels for the establishment of objective indicators of the animal welfare certification standard

**DOI:** 10.5713/ab.21.0079

**Published:** 2021-06-23

**Authors:** Dong Jin Baek, Hyoun Chul Kwon, Ah Lyum Mun, Joo Ri Lim, Sung Won Park, Jin Soo Han

**Affiliations:** 1 Animal Protection and Welfare Division, Animal and Plant Quarantine Agency, Gimcheon, Gyungbuk 39660, Korea; 2 Department of Animal Science, Jeonbuk National University, Jeonju 54896, Korea; 3 Department of Laboratory Animal Medicine and Institute for the 3Rs, College of Veterinary Medicine, Konkuk University, Seoul 05029, Korea

**Keywords:** Animal Welfare, Blood Metabolites, Holstein, Ruminal pH, Rumination Time

## Abstract

**Objective:**

This study was conducted to obtain an objective index that can be quantified and used for establishing an animal welfare certification standard in Korea. For this purpose rumen pH, ruminating time, milk yield, milk quality, and blood components of cows reared in farms feeding high forage level (90%) and farms feeding low forage level (40%) were compared.

**Methods:**

Data on rumen pH, rumination time, milk yield, milk fat ratio, milk protein ratio, and blood metabolism were collected from 12 heads from a welfare farm (forage rate 88.5%) and 13 heads from a conventional farm (forage rate 34.5%) for three days in October 2019.

**Results:**

The rumination time was longer in cattle on the welfare farm than on the conventional farm (p<0.01), but ruminal pH fluctuation was greater in the cattle on conventional farm than the welfare farm (p<0.01). Conventional farms with a high ratio of concentrated feed were higher in average daily milk yield than welfare farms, but milk fat and milk production efficiency (milk fat and milk protein corrected milk/total digestible nutrients) was higher in cattle on welfare farms. Blood test results showed a normal range for both farm types, but concentrations of total cholesterol and non-esterified fatty acid were significantly higher in cows from conventional farms with a high milk yield (p<0.01).

**Conclusion:**

The results of this study confirmed that cows on the animal welfare farm with a high percentage of grass feed had higher milk production efficiency with healthier rumen pH and blood metabolism parameters compared to those on the conventional farm.

## INTRODUCTION

Korea has established certification standards so that farm animals can live normally and maintain their natural behavior. Farms that comply with these standards are certified as animal welfare farms [1]. In ruminants, the production of saliva is activated by rumination and is related to microorganism activity in the rumen, which is directly related to the occurrence of metabolic diseases in ruminants [2].

A high-yield breeding method for Holstein cows has been adopted to increase economic efficiency by greatly increasing the production of milk [3]. However, in high-yield and efficiency cow-orientered feeding management livestock systems, several problems have been reported. These include animal welfare issues and a shortened economic life of the cows, and it was shown that the management of feeding to sustain animal welfare was insufficient [4]. This intensive livestock system in Korea has resulted in, frequently occurring metabolic diseases in dairy cows, such as sub-acute ruminal acidosis [5].

To ensure the health and welfare of ruminant dairy cows, it is important to properly maintain the ruminal environment so that it can function optimally [6]. Ruminants are dependent on rumen microorganisms, which supply most of the nutrients necessary for life and production [7]. For rumen microorganisms to survive, ruminal pH must be properly managed, and this factor is widely used as an indicator of normal rumen functioning [4]. In this regard, the World Animal Health Organization’s cow welfare standard stipulates that normal rumen function should be maintained by providing unlimited amount of grass feed for cows. This will prevent ruminal digestive disorders that are caused by supplying a high percentage of concentrated feed [6] and will comply with animal welfare approved (AWA) dairy cattle standards that require 60% roughage for lactating dairy cows [8]. Korea’s dairy cow animal welfare certification standards also make it mandatory to provide more than 60% of the dry matter as grass feed [1], and this is the main difference between animal welfare farms and conventional farms.

This study aimed to obtain a quantifiable and objective index for establishing welfare standard for Holstein cows in Korea by comparing rumen pH, ruminating time, milk yield, milk quality, and blood components among cows from farms feeding high (welfare farms) and low (conventional farms) forage level.

## MATERIALS AND METHODS

### Animal care

The experimental procedure was approved by the Institutional Animal Care and Use Committee of the Animal and Plant Quarantine Agency (No. 2019-494), Korea.

### Animals and housing

The study was conducted at two dairy cow farms in Ansung City, Gyeonggi Province, Republic of Korea, and 25 dairy cows were selected. The first was an animal welfare certified farm, and the other was a conventional dairy farm in a similar region with, comparable herd size, breeding, and milking systems.

Twelve healthy cows were selected randomly from the welfare certified farm: two primiparous and ten multiparous by parity, five in the early and mid-term (63 to 123 days after delivery), and seven during the late period (220 days or more after delivery) of lactation. Thirteen healthy cows were selected randomly from a conventional farm: six primiparous heads by parity, seven multiparous heads by parity, six heads in the early and mid-term (60 to 100 days after delivery), and 13 heads in the late lactation period (220 days after delivery). The average parity of the two farms was 2.6±1.4 for welfare farm and 2.6±1.7 for the conventional farm. The average number of milking days of the two farms were 176.1±73.6 d for the welfare farm and 180.2±88.7 d for the conventional farm. The average milk yield was 24.8±6.2 kg and 33.9±6.2 kg for the welfare and conventional farms, respectively. The housing facility was an open-type barn structure, and in accordance with the animal welfare certification standards, had a milking cow’s bedding area of 8 m^2^/head and a total area of 16.5 m^2^/head [1].

The shape of the barn was the bedding barn, and the barn was covered with sawdust. To provide comfort to the experimental animals in accordance with the animal welfare standards, a sufficient amount of sawdust was provided to keep the rest area clean and dry.

### Diets

Total mixed ration was used as the experimental feed and cows were fed *ad libitum*. Dry matter intakes were measured by subtracting the remaining amount of feed from the weight of the total amount supplied to each cow. All cow had access to water *ad libitum*.

The ingredients and chemical composition of the diets of both farms are shown in Tables 1 and 2.

The particle size of the feed was measured using a Penn State Particle Size Separator (Table 3).

### Ruminal pH and temperature, and rumination time

To continuously monitor the ruminal pH value, a monitoring sensor (smaXtec PREMIUM sensor, Animal Care Sale GmbH, Graz, Austria) was orally inserted into the rumen using a dedicated insertion gun. Prior to insertion, calibration was performed using pH 4.0 and 7.0 buffer solutions according to the manufacturer’s protocol, and real-time information of pH and temperature was collected through the inserted sensor (length: 12 cm, 3.5 cm in diameter, and 210 g in weight), every 10 min [9]. The rumination time was measured using a collar-attached type sensor, HR-Tag (SCR, Allflex. Netanya, Israel) which was placed on the left side of the neck and rumination data were automatically collected. This logger has a built-in microphone that allows for the recording of the sound of rumination. Time spent ruminating was recorded and stored in 2-h intervals [10].

### Milk yield and composition

Both farms used an automatic robotic milking machine (Lely Astronaut system, Lely Industries N.V., Maassluis, The Netherlands) to collect information on milk yield, and milk fat and protein content. Cows only accessed the automatic robotic milking system associated with their pen. The cows had free access to the automatic robotic milking system 24 h/d. Milking occurred on a voluntary basis, but if an individual cow had not visited the automatic robotic milking system for over12 h, she was fetched by farm staff to be milked [11].

### Blood sampling and analysis

Blood samples were collected from each experimental cow 4 to 5 h after the 7 o’clock morning feed. Blood was drawn from the coccygeal vein of 12 cows from the certified welfare farm and 13 cows from the conventional farm.

The samples were placed in a blood collection tube (VACUETTE serum clot, 9 mL) and serum was separated by centrifugation (1,500×g at 4°C for 5 min). Serum samples were tested for alanine aminotransferase, aspartate aminotransferase, total bilirubin, and total cholesterol levels using a serum biochemical analyzer (Beckman Coulter AU480; Beckman Coulter, Inc., Brea, CA, USA). Total protein, albumin, albumin/globulin ratio, glucose, blood urea nitrogen (BUN), creatine, Ca, P, Mg, and non-esterified fatty acid (NEFA) levels were also analyzed.

### Statistical analysis

Data were analyzed using the MIXED procedure in the SAS package program (SAS Inst. Inc., Cary, NC, USA) as a completely randomized design. The experimental model was as follows:


Yi(t)=μ+Ti+Ei(t)

where μ is the average value, Ti is the treatment value and Ei(t) is the error value. The fixed-effect of the welfare farm and random effects were not considered. Least squares mean was assessed using pairwise comparisons and the orthogonal contrast method. Statistical differences and tendencies were accepted at p-values less than 0.05 and 0.10, respectively. All means are presented as the least square means.

## RESULTS

### Ruminal pH and temperature, and rumination time

**Figure 1:**
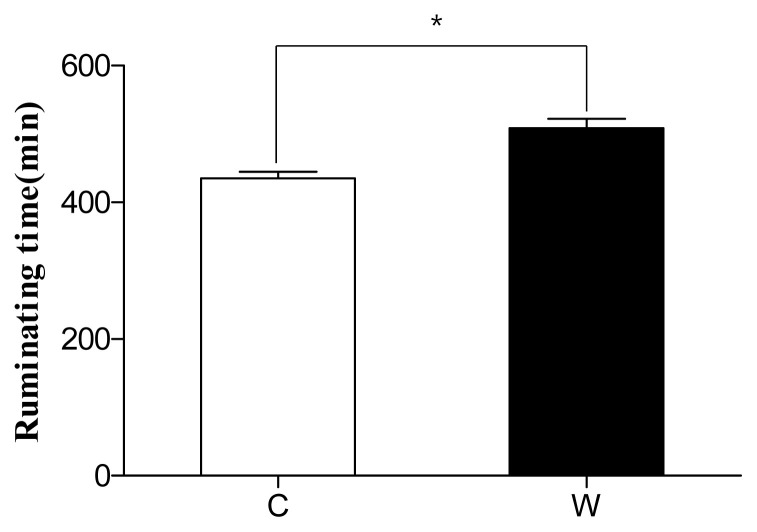
Comparison of ruminating time between conventional farm and welfare farm over 3 days. C, conventional farm; W, welfare farm. * Indicates significant differences between conventional and welfare farms (p<0.01).

The average ruminal temperature of all the cows from both farms was 39°C±1°C, and the daily average ruminal pH was 6.2±0.2 for the welfare farm and 6.3±0.2 for the conventional farm. The lowest daily pH values from the welfare and conventional farms were 5.9±0.2 and 5.8±0.2 (p<0.05), and the highest pH values were 6.5±0.2 and 6.7±0.2 (p<0.01), respectively. The pH fluctuation of the two farms was 0.6±0.2 and 0.9±0.2 (p<0.01), respectively (Table 2). The average rumination time was 508.84±73.8 min for the welfare farm and 435.4±50.5 min for the conventional farm (p<0.01) (Figure 1). The average daily visit to drinking boals during three days was 5.9±1.7 and 6.7±1.9 times, respectively (Table 4).

### Milk yield and composition

During the three days experiment, the average milk production was 24.0±6.3 kg/d in the welfare certified farm and 33.9± 6.1 kg/d in the conventional farm (p<0.01). Milk fat content was 4.2%±0.5% in the welfare certified farm (p<0.01) and 3.8%±0.5% in the conventional farm, and the milk protein contents were 3.2%±0.2% (p<0.01) and 3.4%±0.2% for the welfare and conventional farms respectively. Fat-and milk protein-corrected milk yield (FPCM) was 24.2 kg/d in on the welfare farm, and 33.4 kg/d on the conventional farms (p<0.01). Milk production efficiency (FPCM/TDN) was 1.8±0.5 and 1.7±0.3 for the two farms, respectively (Table 5).

### Blood parameters

Blood parameters of cows from both farms were within the normal range as obtained from other reported studies except total cholesterol, albumin, glucose, BUN, creatinine levels, and the A/G ratio, which were significantly higher in cows on conventional farms than in those on welfare farms (p< 0.05), and total protein values of cows on the welfare farm were significantly higher than of those on the conventional farm (p<0.01) (Table 6).

## DISCUSSION

Recently, the importance of performing dairy cattle welfare assessment using animal-based measurement was emphasized by the World Animal Health Organization [12,13] and major criteria for welfare assessment were measured in this study.

In this study, the average rumination time of cows on the welfare farm was 508.8±73.8 min, which was significantly longer than that of cows on the conventional farm which was 435.4±50.5 min. This is similar to results of previous studies on welfare farms that provide with a high percentage of grass feed to their cattle [14–16].

Originally, we expected that the welfare farm that fed a high percentage of grass to its cows, would maintain a relatively high pH, compared to that of the conventional farm [15,16]. However, a significant difference in rumen pH was not found between the two farms. This is unexpected results because it is generally accepted that higher roughage feeding results in higher ruminal pH with more ruminations [17]. Current results of present study might be related to differences in particle size of feeds offered to animals of two farms. The forage used in the conventional farm was longer than that used in the welfare farm, which affected ruminal pH. It was concluded that the particle size of the feed grain on the welfare farm had an effect on the decreased rumen pH [18].

However, unlike the comparison of the overall average pH on the two farms, the average daily pH change, calculated from the difference between the maximum and minimum pH values, was significantly different between the two farms; 0.6±0.2 for the welfare farm and 0.9±0.2 for the conventional farms, which indicates that higher forage promotes much more stable ruminal pH environment and hence provides more favorable condition for rumen microbial growth. These results are similar to those of previous studies in which the lower the proportion of grass feed, the wider the range of changes in pH was observed [19]. This suggests that pH is relatively stable in the rumen of cows on welfare farms [20].

The frequency of drinking water was found to be higher on the conventional farm than on the welfare farm. This might be due to the increase in osmolarity of the rumen content of higher concentrate and is attributed to the regulation of pH homeostasis in the rumen of cows on conventional farms where the range of pH changes in the rumen was large, and previous goat research supports this finding [21].

Therefore, it can be concluded that the welfare farm, which had a relatively high percentage of grass feed, showed positive results as compared to the conventional farm with respect to the rumen state.

The average daily milk yield of cows from the welfare farm was 9 kg higher than those from the conventional farm. However, the milk production efficiency (a value obtained by dividing the corrected milk fat milk protein flow rate by the plasticized total nutrients) was 1.8±0.5 for the welfare farm and 1.7±0.3 for the conventional farm, showing a higher production efficiency of the welfare farm. Current result is expected one and high energy diets would promote higher milk yield, but the same diet is not always superior in milk production efficiency [22].

The results of the blood analysis confirmed that there were no major differences in most of parameters among cows from both farms and those values were within the normal range, as shown in previous studies that measured the same blood component [23]. However, blood components related to energy balance, such as total cholesterol, total protein, BUN, and NEFA, were significantly different between the two farms. This implies that feeding different levels of forages influenced metabolic rates of cows and resulted in high milk yields in conventional farms, which provided high-energy feed to cows [24–26] This may in turn, have a negative impact on the economic lifespan of cows by exerting a high metabolic burden [27,28].

In summary the level of forage affected rumen pH, milk production characteristics, and blood metabolism components and these results may provide some useful index in establishing the guideline for welfare standard in Korea. Further research by using more animals with different types and levels of forage sources under various environmental conditions are warranted to provide more valid criteria for the animal welfare certification policy system in Korea.

## Figures and Tables

**Table 1:** Chemical composition of the diet on the conventional and welfare farms

Items	TMR		Concentrate	
	
	C^1)^	W^1)^	C^1)^	W^1)^
Ingredients (kg/head)				
Beet pulp	2.50	-	-	-
Cotton seed whole	2.50	-	-	-
Alfalfa hay	2.17	4.00	-	-
Timothy hay	2.17	-	-	-
Oats hay	3.67	4.00	-	-
Tall fascue strew	2.17	-	-	-
Meadow hay	-	4.00	-	-
Algoie grass hay	-	12.00	-	-
Concentrate feed	-	-	14.17	3.00
Vit and Min premix	0.12	-	-	-
Chemical composition (% DM)				
CP	13.47	13.41	21.05	19.59
NDF	57.10	61.49	14.20	18.31
eNDF	47.81	55.66	0.45	1.15
NSC^2)^	19.33	18.51	44.04	43.11
Energy content				
TDN (% DM)	68.20	59.06	74.33	73.86
NE (Mcal/kg)	2.36	2.04	2.68	2.67
NEm (Mcal/kg)	1.48	1.19	1.82	1.80
NEg (Mcal/kg)	1.54	1.32	1.74	1.73
NEl (Mcal/kg)	0.89	0.62	1.24	1.22

TMR, total mixed ration; DM, dry matter; CP, crude protein; NDF, neutral detergent fiber; eNDF, effective neutral detergent fiber; NSC, nonstructural carbohydrate; TDN, total digestible nutrients; NE, net energy; NEm, net energy for maintenance; NEg, net energy for grain; NEl, net energy for lactation.

1) C, conventional farm; W, welfare farm.

2) NSC = 100–(CP+NDF+Fat+Ash+pectin).

**Table 2:** Feed intake and nutrient composition of experimental animals

Item	C^1)^	W^1)^
DMI (kg/d)	27.74	21.19
Forage ratio (%)	34.54	88.46
NDF (kg/d)	9.96	8.39
NSC (kg/d)	6.76	4.93
TDN (kg/d)	19.80	13.32
NEl (Mcal/d)	451	301
Forage DMI (kg/d)	9.57	18.74
Concentrate DMI (kg/d)	18.17	2.45

DMI, dry matter intake; NDF, neutral detergent fiber; NSC, nonstructural carbohydrate; TDN, total digestible nutrients; NEl, net energy for lactation.

1) C, conventional farm; W, welfare farm.

**Table 3:** The measurement of the particle size of total mixed ration

	Item	C^1)^ (g)	W^1)^ (g)	C^1)^ (%)	W^1)^ (%)
1 sieve	Upper sieve (1.905 cm)	218	162	43.6	32.4
2 sieve	Middle sieve (0.7874 cm)	63	76	12.6	15.2
3 sieve	Lower sieve (0.4604 cm)	63	97	12.6	19.4
4 sieve	Bottom pan (<0.4604 cm)	156	165	31.2	33
	Total	500	500	100	100

C, conventional farm; W, welfare farm.

**Table 4:** Comparison of ruminal pH value between conventional farm and welfare farm for 3 days

Item	C^1)^	W^1)^	p-value
Mean pH	6.25±0.16	6.24±0.21	0.81
Min pH	5.79±0.22	5.90±0.23	<0.05
Max pH	6.67±0.18	6.53±0.23	<0.01
Max-min pH	0.87±0.21	0.62±0.16	<0.01
Drinking frequency(No)	5.9±1.7	6.7±1.9	-

1) C, conventional farm; W, welfare farm.

**Table 5:** Comparison of lactation performance between conventional farm and welfare farm for 3 days

Items	C^1)^	W^1)^	p-value
Milk yield (kg/d)	33.94±6.06	24.03±6.25	<0.01
Milk fat (%)	3.84±0.45	4.15±0.48	<0.01
Milk protein (%)	3.37±0.17	3.19±0.16	<0.01
FPCM	33.42±6.23	24.21±6.06	<0.01
FPCM/TDN	1.68±0.32	1.82±0.45	0.17

FPCM, fat protein correction milk; FPCM/TDN, fat protein correction milk/total digestible nutrients.

1) C, conventional farm; W, welfare farm.

**Table 6:** Comparison of metabolic profiles between conventional farm and welfare farm for 3 days

Item	C^1)^	W^1)^	p-value
ALT (U/L)	35.64±5.79	33.72±12.98	0.65
AST (U/L)	86.18±16.75	94.28±10.98	0.19
T-BIL (mg/dL)	0.23±0.05	0.22±0.07	0.73
T-Cholesterol (mg/dL)	276.38±66.10	198.75±35.96	<0.05
T-Protein (mg/dL)	7.29±0.34	8.01±0.16	<0.01
Albumin (g/dL)	3.42±0.15	3.54±0.04	0.07
A/G, ratio	0.89±0.08	0.80±0.08	<0.05
Glucose (mg/dL)	61.48±4.17	43.67±4.54	<0.01
BUN (mg/dL)	15.77±1.54	12.11±1.53	<0.01
Creatine (mg/dL)	0.93±0.08	0.84±0.06	<0.01
Ca (mg/dL)	9.80±0.45	9.67±0.52	0.55
P (mg/dL)	6.11±0.87	6.47±0.18	0.33
Mg (mg/dL)	2.65±0.21	2.45±0.17	<0.05
NEFA (uEq/L)	151.7±25.69	109.2±96.78	<0.01

ALT, alanine aminotransferase; AST, aspartate aminotransferase; T-BIL, total bilirubin; T-Cholesterol, total cholesterol; T-Protein, total protein; A/G, ratio, albumin/globulin ratio; BUN, blood urea nitrogen; Ca, calcium; P, phosphorus; Mg, magnesium; NEFA, non-esterified fatty acid.

1) C, conventional farm; W, welfare farm.
